# Preclinical Immunogenicity and Efficacy of Optimized O25b O-Antigen Glycoconjugates To Prevent MDR ST131 E. coli Infections

**DOI:** 10.1128/iai.00022-22

**Published:** 2022-03-21

**Authors:** Laurent Chorro, Zhenghui Li, Ling Chu, Suddham Singh, Jianxin Gu, Jin-hwan Kim, Kaushik Dutta, Rosalind Pan, Srinivas Kodali, Duston Ndreu, Axay Patel, Julio C. Hawkins, Chris Ponce, Natalie Silmon de Monerri, David Keeney, Arthur Illenberger, C. Hal Jones, Lubomira Andrew, Jason Lotvin, A. Krishna Prasad, Isis Kanevsky, Kathrin U. Jansen, Annaliesa S. Anderson, Robert G. K. Donald

**Affiliations:** a Pfizer Vaccine Research and Development, Pearl River, New York, USA; b Citranvi Biosciences, Chapel Hill, North Carolina, USA; Washington State University

**Keywords:** *Escherichia coli*, ST131, glycoconjugate vaccine, O antigen, O25b, MDR

## Abstract

Multivalent O-antigen polysaccharide glycoconjugate vaccines are under development to prevent invasive infections caused by pathogenic *Enterobacteriaceae*. Sequence type 131 (ST131) Escherichia coli of serotype O25b has emerged as the predominant lineage causing invasive multidrug-resistant extraintestinal pathogenic E. coli (ExPEC) infections. We observed the prevalence of E. coli O25b ST131 among a contemporary collection of isolates from U.S. bloodstream infections from 2013 to 2016 (*n* = 444) and global urinary tract infections from 2014 to 2017 (*n* = 102) to be 25% and 24%, respectively. To maximize immunogenicity of the serotype O25b O antigen, we investigated glycoconjugate properties, including CRM_197_ carrier protein cross-linking (single-end versus cross-linked “lattice”) and conjugation chemistry (reductive amination chemistry in dimethyl sulfoxide [RAC/DMSO] versus ((2-((2-oxoethyl)thio)ethyl)carbamate [eTEC] linker). Using opsonophagocytic assays (OPAs) to measure serum functional antibody responses to vaccination, we observed that higher-molecular-mass O25b long-chain lattice conjugates showed improved immunogenicity in mice compared with long- or short-chain O antigens conjugated via single-end attachment. The lattice conjugates protected mice from lethal challenge with acapsular O25b ST131 strains as well as against hypervirulent O25b isolates expressing K5 or K100 capsular polysaccharides. A single 1-μg dose of long-chain O25b lattice conjugate constructed with both chemistries also elicited robust serum IgG and OPA responses in cynomolgus macaques. Our findings show that key properties of the O-antigen carrier protein conjugate such as saccharide epitope density and degree of intermolecular cross-linking can significantly enhance functional immunogenicity.

## INTRODUCTION

Extraintestinal pathogenic Escherichia coli (ExPEC) strains are a leading cause of a wide range of invasive infections affecting all age groups and incurring a substantial economic burden (1–5). Escherichia coli sequence type 131 (ST131) serotype O25b has emerged as a dominant worldwide pandemic ExPEC clone, causing predominantly community-onset bloodstream and urinary tract infections (UTIs) with high rates of resistance to extended-spectrum β-lactamase (ESBLs) and fluoroquinolones (6, 7). E. coli O25b ST131 has become the most prevalent multidrug-resistant (MDR) lineage due to the sequential acquisition of genes associated with virulence and antibiotic resistance (8, 9) together with its success as an intestinal colonizer and propensity for fecal-oral transmission (10, 11).

Vaccines offer an alternative approach to combating hard-to-treat MDR E. coli. The serotype O25b O antigen is well validated as a therapeutic target of bactericidal antibodies in preclinical models. Passive immunization with monoclonal antibodies (MAbs) specific for epitopes recognizing the O25b O-antigen polysaccharide repeat unit were sufficient to protect against lethal challenge with MDR O25b ST131 isolates (12, 13). One of these MAbs showed multiple mechanisms of action: namely, direct complement-mediated and opsonophagocytic killing as well as endotoxin neutralization in respective lethal bacteremia and endotoxemia challenge models (12, 14). Multivalent O-antigen-based prophylactic vaccines capable of eliciting protective polyclonal antibodies to prevent invasive E. coli disease have been under investigation since the 1990s. Native O antigens can be cleaved from bacterial lipopolysaccharide (LPS) by acid hydrolysis, and isolated E. coli O antigens have been conjugated to the Pseudomonas exotoxin A (EPA) carrier protein by chemical cross-linking (15). Alternatively, a bioconjugation platform was developed allowing conjugation of O antigens to EPA using a recombinant E. coli platform (16, 17). In this case, the O-polysaccharide is assembled on its carrier lipid and enzymatically transferred to specific residues of the protein carrier via an N-glycosidic linkage (18). A potential limitation of the bioconjugation strategy is that it leaves little room for further optimization of the glycoconjugate antigen, for example, to increase the density of functional antibody epitopes or the ratio of polysaccharide antigen to carrier protein.

A confounding aspect in the development of prophylactic multivalent vaccines has been the variable immunogenicity observed for some O-antigen serotypes. In particular, O antigens of the E. coli O25 serotype, which include O25a and O25b subtypes, have elicited weaker polyclonal antibody responses than other serotypes in preclinical models (15, 16). Serotype O25a and O25b glycoconjugates have also been relatively poor immunogens in human volunteers compared with other serotypes (19–21). As a mitigation, Janssen, for example implemented a compensatory 2-fold increase in dose of their O25b bioconjugate relative to the other O-antigen conjugates in a subsequent phase II study with their ExPEC4V vaccine (22).

The goal of this study was to select an immunogenic O25b glycoconjugate as a major component of a multivalent O-antigen vaccine to prevent invasive ExPEC. To identify the O25b O-antigen conjugate most capable of eliciting a strong functional antibody response in preclinical models, we evaluated a variety of conjugation strategies linking O25b O antigen to CRM_197_ carrier protein. We found that lattice conjugates provide superior immunogenicity and demonstrate for the first time that active immunization can protect mice from lethal challenge with invasive MDR ST131 E. coli. We also observed robust functional antibody response in immunized nonhuman primates (NHPs).

## RESULTS

### Invasive isolate surveillance and predominance of the O25b O-antigen serotype.

**FIG 1:**
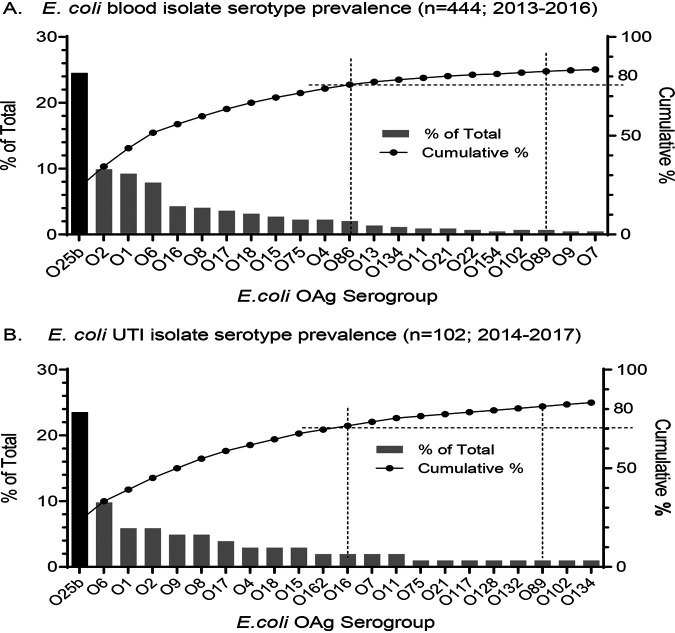
The O25b serotype is predominant among contemporary invasive BSI and UTI isolates. (A) A total of 444 U.S. blood isolates (2013 to 2016) and (B) 102 globally sourced isolates from UTI bladder, kidney, ureter, and urethra infections (2014 to 2016) were examined. Dotted lines show that 12-valent or 20-valent O-antigen vaccines would provide theoretical vaccine coverage of ≥70% and >80%, respectively.

E. coli isolates causing bloodstream infections (BSIs) and UTIs were obtained from the Antimicrobial Testing Leadership and Surveillance (ATLAS; https://atlas-surveillance.com/) program. E. coli BSI isolates (*n* = 444) from 2013 to 2016 were obtained from hospitals in the ATLAS network representing 17 U.S. states. Of these isolates, 43% were from patients >65 years of age and 9.2% from infants <1 year of age. E. coli UTI isolates corresponding to kidney, ureter, urethra, and bladder infections from 2014 to 2017 were selected (*n* = 102) to avoid the inadvertent sampling of contaminating commensal isolates that are common in urine cultures. Due to the stringency of the infection site selection (which reduced overall numbers), the sampling of these UTI isolates included strains from North America (16.5%), Europe (71.8%), South America (4.9%), and Asia (6.8%). In this case, the proportions of isolates from elderly and infant patients were 53% and 5%, respectively. *In silico* serotyping of O antigens and other genotypic information was determined through the analysis of whole-genome sequence data. The prevalences of the O25b serotype were 25% (112/444) in the BSI collection and 24% (24/102) in the UTI isolate collection (Fig. 1); 95% (107/112) of the O25b BSI and 100% (24/24) of the O25b UTI isolates belong to the same prevalent clonal ST131 sublineage harboring H4 (*fliC*) and H30 (*fimH*) alleles (23). A minor subset (4%; 5/112) of the O25b BSI strains were ST69 and carried the H18 (*fliC*) and H27 (*fimH*) alleles. Based on ATLAS MIC panel data, rates of resistance to the fluoroquinolone antibiotics ciprofloxacin or levofloxacin among the ST131 O25b isolates were 93% and 88% for BSI and UTI strains, respectively. The frequencies of resistance to third-generation cephalosporins ceftazidime and ceftriaxone were 33% for the O25b ST131 BSI strains and 36% for the O25b ST131 UTI strains. Only a single serotype O25a strain, with genotype ST73:H1(*fliC*):H12(*fimH*), was identified, confirming the dominance of the O25b subtype among these contemporary clinical strains. After serotype O25b, the distribution of the next 10 most prevalent O-antigen serotypes was largely shared between the BSI and UTI strain collections and included serotypes O1a, O2, O6, O8, O16, O17, O18, O15, O75, and O4 (Fig. 1). Notable exceptions were serotypes O86 (absent from the UTI collection), O162 (absent from the BSI collection), and O9, which was considerably more prevalent among UTI strains than BSI strains.

### Expression of O25b long-chain O antigens in E. coli.

**FIG 2:**
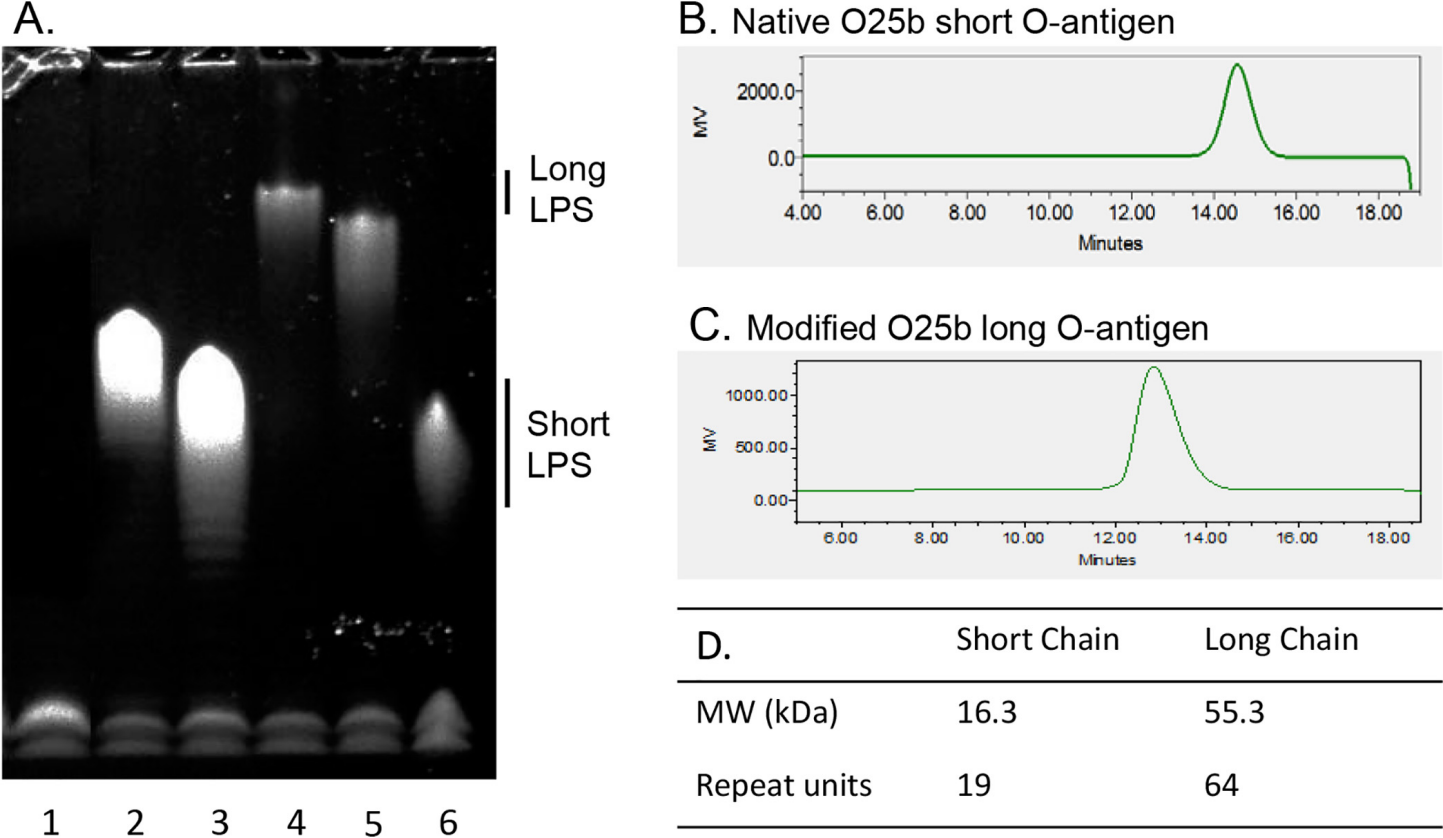
Salmonella *wzzB* or *fepE* genes expressed from a plasmid can extend O-antigen chain length in an E. coli Δ*wzzB* strain. (A) LPS extracted from an O25b *wzzB* knockout strain and plasmid transformants were resolved by SDS-PAGE. Lane 1, untransformed O25b Δ*wzzB* parent; lane 2, Salmonella *wzzB*; lane 3, E. coli *wzzB*; lane 4, Salmonella *fepE*; lane 5, E. coli *fepE*; lane 6, E. coli LPS control. (B and C) Analytical size exclusion chromatography (SEC) profiles of purified native short and Salmonella *fepE*-induced long O antigens and (D) their properties.

To facilitate development of scalable purification and conjugation methods and to increase the density of O25b O-antigen functional epitopes, we explored increasing the length of the polysaccharide repeat unit by genetic manipulation of serotype O25b E. coli strains. This was accomplished by genetically complementing a deletion of the endogenous chain length regulator *wzzB* with a plasmid-borne copy of the heterologous Salmonella enterica serovar Typhimurium *fepE* gene, which confers longer O-antigen chain length in Salmonella (24, 25). SDS-PAGE profiles of LPS extracted from E. coli strains harboring *wzzB-* or *fepE-*containing plasmids confirm that chain length is determined by the chain length regulator expressed. Accordingly, as shown in Fig. 2A, expression of Salmonella or E. coli *fepE* genes resulted in substantially longer LPS than expression of corresponding *wzzB* genes that yielded shorter-chain LPS typical of native E. coli. Salmonella *fepE* was selected for further investigation as it generated the longest chain length of all *fepE* variants investigated. Bacterial cultures were treated with acetic acid under high heat to selectively cleave the O antigen from lipid A at the labile 2-keto-3-deoxyoctanoic acid (KDO) linkage present at the reducing end terminus of the LPS inner core oligosaccharide. The released polysaccharides were purified and characterized. Compared with O antigen purified from a native O25b strain, the O antigen obtained from the recombinant strain expressing Salmonella *fepE* was 3.4 times larger, with the number of repeat units increased from 19 to 64 (Fig. 2B to D). The structure of the purified O25b long O antigen was determined by nuclear magnetic resonance (NMR) analysis and confirmed to match the published structure for native short-chain O antigen (13) by plotting the chemical shift difference between the sugar resonances of the O25b long O antigen and the single repeat unit attached to the outer core oligosaccharide (see Fig. S1 in the supplemental material). The difference in the resonance assignment seen in the residue C can be attributed to the different structure that was analyzed in the published paper (13). In this case, the authors used a smaller oligosaccharide which has just one repeat unit attached to the core sugar units. As a result, there is no repeat unit linked to the residue C. Furthermore, residue E is linked to the outer core sugar units. Thus, there will be some chemical shift differences seen in residue C and residue E as their chemical environment is altered compared to the repeat unit present in the O25b long O-antigen polysaccharide. These changes are highlighted in the structure (Fig. S1). Sequential connectivities between the sugars were confirmed using long-range ^1^H-^13^C heteronuclear multiple-bond correlations (HMBC). The α- and β-glycosidic linkages were determined using the ^1^J_CH_ correlations.

### Construction of O25b O-antigen glycoconjugates.

**FIG 3:**
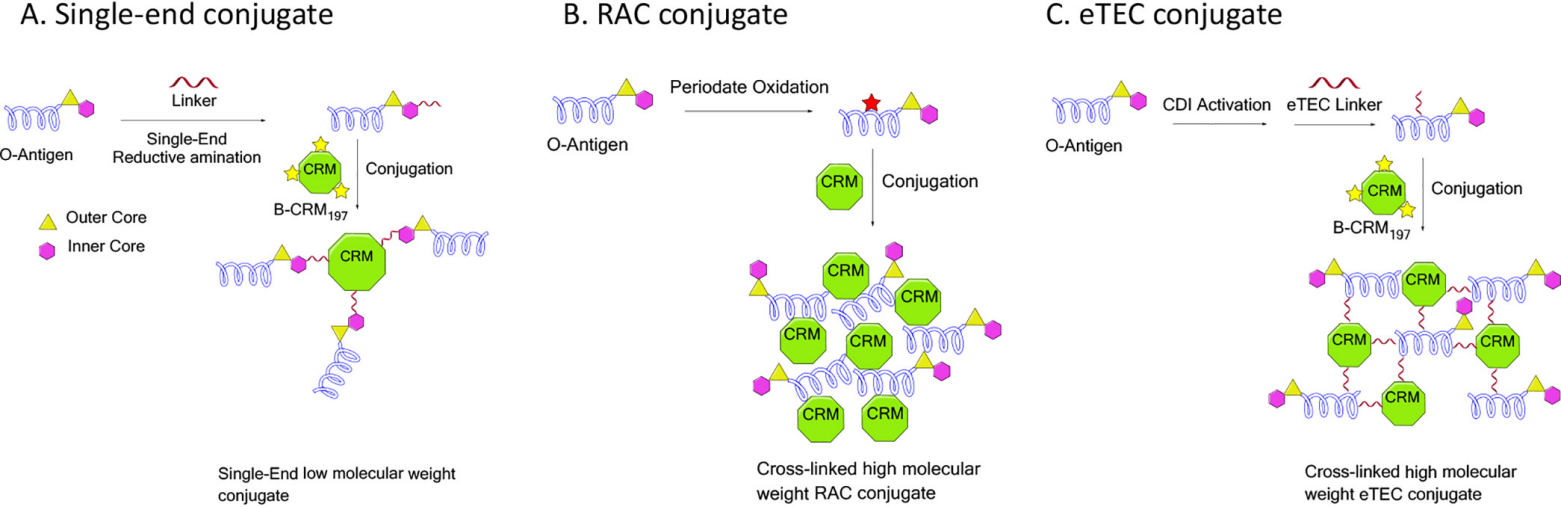
Illustration of glycoconjugate chemistry strategies for (A) single-end conjugate, (B) reductive amination conjugate (RAC), and (C) 2-((2-oxoethyl)thio)ethyl carbamate (eTEC) conjugate. Properties are described in Table S1. B-CRM_197_ is bromo-activated CRM_197_. CDI is 1,1′-carbonyldiimidazole.

Purified O25b O antigens were chemically conjugated to CRM_197_ carrier protein using the three different methods illustrated in Fig. 3. Using a linker-mediated directional coupling strategy, single-end conjugates with native short or engineered long-chain O antigens were constructed to create “sun”-type glycoconjugates. In this case, the terminal KDO of the O antigen was derivatized with a disulfide amine linker, which subsequently is reduced to liberate a free sulfhydryl and conjugated to activated carrier protein via formation of a thioether bond between the saccharide linker and the bromo moiety on the protein. Long-chain O antigens were also conjugated to CRM_197_ by random intermolecular cross-linking to generate higher-molecular-mass soluble aggregate or lattice glycoconjugates. Here, reductive amination chemistry in DMSO (RAC/DMSO) or (2-((2-oxoethyl)thio)ethyl)carbamate (eTEC) linker chemistries were used. For the RAC/DMSO process, the carbohydrate was activated by periodate oxidation followed by RAC conjugation. The properties and quality attributes of the O25b single-end and lattice conjugates generated are summarized in Table S1 in the supplemental material. The output molar saccharide/carrier protein ratio achieved with the short-chain single-end CRM_197_ conjugate was 3:1 (0.7 by mass), while for the long-chain single-end CRM_197_ conjugate, a 1:1 ratio (0.9 by mass) was obtained. Three different eTEC lattice conjugates were generated, differing in their degree of cross-linking, conjugate size, and yield, all of which increased with the level of disulfide linker derivatization.

### Detection of anti-O25b O-antigen antibodies in mouse sera.

**FIG 4:**
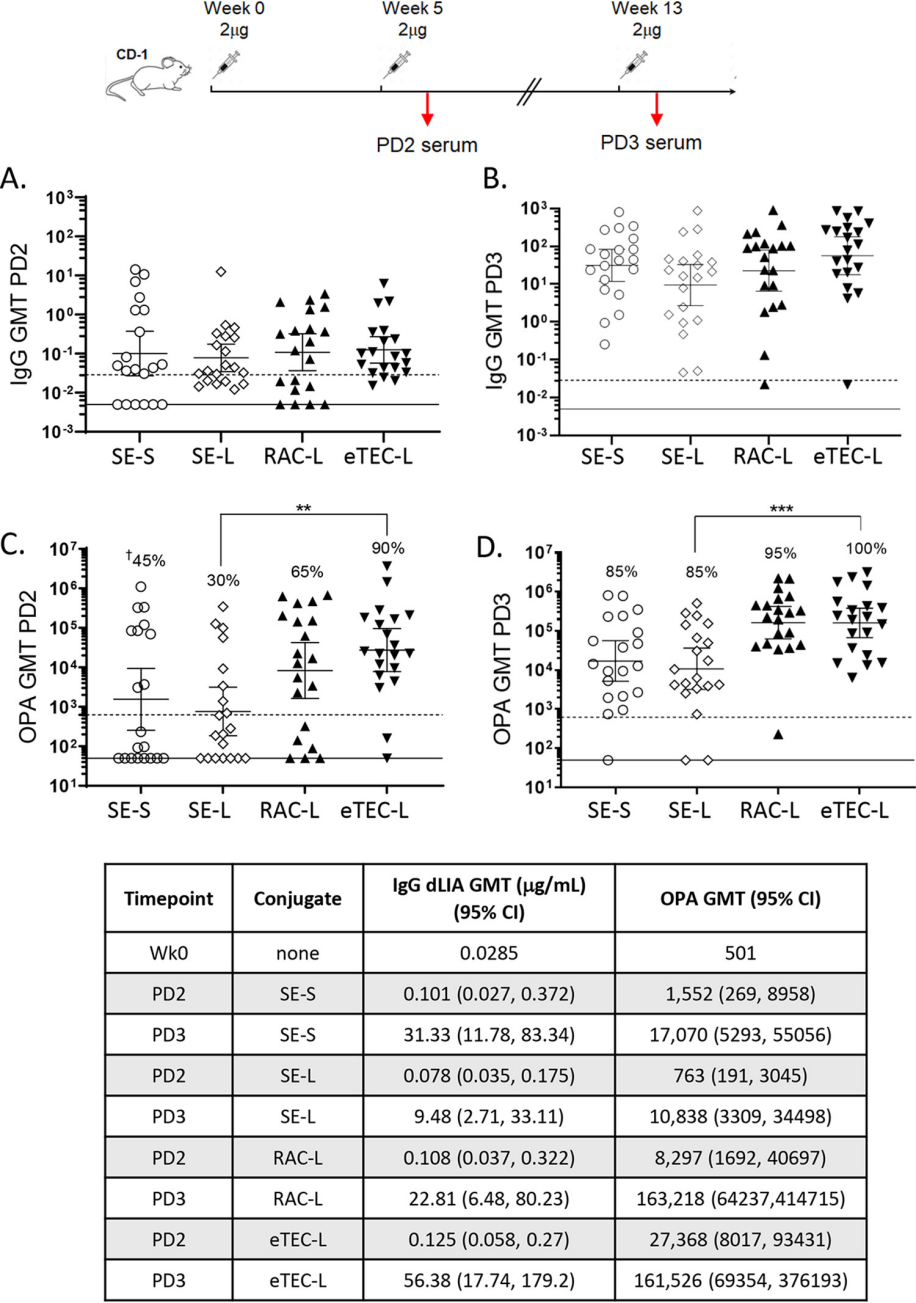
Three doses of O25b conjugates are required to generate robust and uniform IgG and OPA responses in CD-1 mice. The dosing and bleed schedule are illustrated. (A, C) Postdose 2 (PD2) IgG and OPA geometric mean titers (GMTs). (B, D) Postdose 3 (PD3) IgG and OPA GMTs. Long-chain RAC/DMSO (RAC-L) and long-chain eTEC (eTEC-L) lattice conjugates (closed symbols) yield higher OPA responses than the single-end long (SE-L) or single-end short (SE-S) conjugate (open symbols). OPA data were generated with MDR O25b strain PFEEC0068 with a ratio of HL60 effector cells to bacteria of 100:1. The eTEC conjugate was generated using intermediate levels of linker thiol activation (10%) compared with other eTEC conjugates (Table S1). Responder rates are indicated as percentages (marked with dagger), and statistically significant differences are marked with asterisks (***, *P* < 0.001; **, *P* < 0.05). The dotted line is the unvaccinated mouse baseline titer (*n* = 20); the solid line is the 1/2× limit of detection (LOD) value of 50. GMT values with 95% confidence intervals (CI) are shown in the table.

Serum antigenicity of the high-molecular-mass RAC/DMSO and eTEC lattice conjugates was directly compared with single-end short and single-end long O-antigen conjugates (Fig. 4). Groups of 20 CD-1 mice were dosed by subcutaneous (s.c.) injection with 2 μg of glycoconjugate at weeks 0, 5 and 13, with bleeds taken after the first and second boosts. Levels of antigen-specific IgG were determined by quantitative Luminex assay with an O25b-specific anti-mouse MAb as an internal standard. Baseline IgG levels were determined in serum pooled from 20 randomly selected unvaccinated mice. A dose of 2 μg of free unconjugated O25b long-chain polysaccharide was unable to induce levels of antigen-specific IgG that were significantly higher than those of unvaccinated mice at any time point (data not shown). In contrast, robust IgG responses were observed for all glycoconjugates after the second glycoconjugate boost (postdose 3 [PD3]), while intermediate more variable IgG responses were observed after the first boost (postdose 2 [PD2]). The IgG responses to the four O25b glycoconjugates were not significantly different from each other at either time point. However, functional antibody titers and responder rates determined by opsonophagocytic assays (OPAs) were consistently higher for the long-chain RAC/DMSO and eTEC lattice conjugates than the responses observed with the single-end short- or single-end long-chain glycoconjugates.

Sera from the intermediate PD2 time point were tested in the O25b OPA to assess the influence of aluminum phosphate (AlPO_4_) adjuvant on the functional response to the O25b long RAC/DMSO O-antigen conjugate at 0.2-μg and 2-μg doses. The AlPO_4_ formulation improved OPA responder rates and increased geometric mean titers (GMTs) at both dose levels (see Fig. S2 in the supplemental material). OPA responses were also used to identify the level of chemical activation conferring optimal immunogenicity of long O-antigen eTEC conjugates. Higher and lower levels of O-antigen activation than the default level (10%) were evaluated in a separate mouse immunogenicity study (17% versus 4% mol of thiol/mol of polysaccharide). Higher levels of activation increased yield and molecular mass of the antigens (Table S1) and significantly improved titers and responder rates when OPA titers from both dose levels for the highest versus lowest (17% versus 4%) levels of O-antigen activation are compared (Fig. S2B).

### ST131 O25b isolate virulence and protection from lethal challenge following glycoconjugate vaccination.

To establish a vaccine protection model, we characterized four MDR O25b ST131 blood isolates by genotypic and phenotypic analyses (Table 1). All four strains contained genetic resistance determinants consistent with ATLAS antibiotic MIC panel data (see Fig. S3 in the supplemental material). Three strains were additionally resistant to fluoroquinolone antibiotics (PFEEC0065, PFEEC0066, and PFEEC0068), and two were resistant to trimethoprim-sulfamethoxazole (PFEEC0066 and PFEEC0068). Profiling of 40 established E. coli virulence factor genes revealed that the four strains were highly similar to reference strain O25b ST131 EC958 (26) (see Fig. S4 in the supplemental material). The sole exception was the absence of the *yehA* adhesin gene in EC598 and PFEEC0065. Two of the strains were genotypically and phenotypically unencapsulated, while two others expressed heat-labile group II class K5 and K100 capsular polysaccharides. When grown in nutrient-rich Luria broth (LB), capsule expression masked underlying O antigens, while growth in the mammalian cell culture medium Dulbecco’s modified Eagle’s medium (DMEM) reduced capsule expression, exposing the O antigens (see Fig. S5 in the supplemental material). Under these conditions, the encapsulated K5 and K100 strains were highly susceptible to killing *in vitro* by O25b rabbit immune sera (see Fig. S6 in the supplemental material).

**TABLE 1:** Properties of MDR O25b BSI isolates evaluated in OPAs and in mouse lethal challenge models

Isolate (O25b ST131)	Source	Drug resistance^*a*^	Presence of group II K-CPS genes	K-CPS phenotype^*b*^	Challenge dose i.p. (CFU/animal)
PFEEC0102	US, 2003	ESBL	No (K^−^)	CPS^−^	2.0 × 10^8^
PFEEC0068	US, 2006	Imp, FQ, SXT	No (K^−^)	CPS^−^	1.6 × 10^8^
PFEEC0066	Morocco, 2016	ESBL, FQ, SXT	Yes (K5)	CPS^+^	5.0 × 10^6^
PFEEC0065	Czech Republic, 2016	Amp, FQ	Yes (K100)	CPS^+^	5.0 × 10^6^

a ESBL, extended-spectrum β-lactamase (resistance to cephalosporins); FQ, fluoroquinolone; Imp, imipenem; SXT, trimethoprim/sulfamethoxazole; Amp, ampicillin.

b Heat-sensitive masking of O antigen by K-CPS in LB-grown cells.

Virulence of the four ST131 MDR strains was assessed in CD-1 mice following intraperitoneal (i.p.) and intravenous (i.v.) challenge (see Fig. S7 in the supplemental material). Dose ranging studies for each strain identified a bacterial inoculum resulting in 20% survival level, the target control threshold for vaccine protection experiments; this baseline level provides a more robust consistency when comparing vaccine efficacy across bacterial strains compared with 0% survival in sham-treated groups, where the rapid onset of mortality may preclude accurate scoring and humane euthanasia. The two unencapsulated strains required 40- to 100-fold more live bacteria to achieve the same level of lethality by i.p. challenge than the encapsulated K5 and K100 strains (Table 1; Fig. S7A). As the virulence gene profiles of these strains are otherwise nearly identical, it seems likely that K5 and K100 capsule expression contributes to the enhanced virulence phenotype of these isolates. The O25b:K5 challenge strain was determined to also be lethal via i.v. administration, albeit at higher dose levels. For this strain, the i.v. challenge dose conferring 20% survival was 1 × 10^8^ CFU, compared with 5 × 10^6^ CFU for the i.p. route (Fig. S7B).

**FIG 5:**
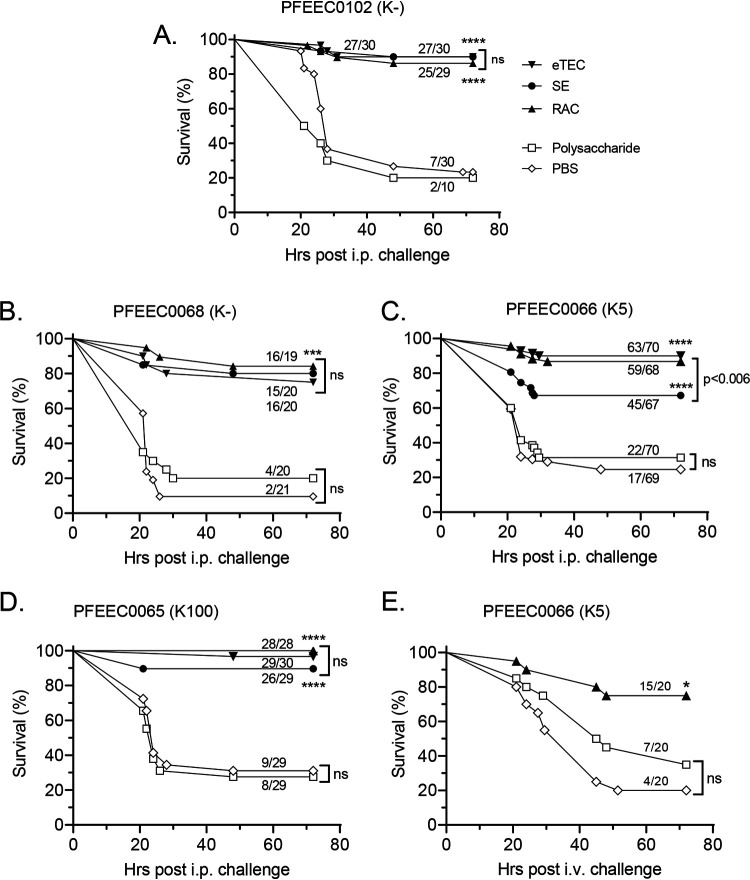
Protection by O25b glycoconjugates against lethal challenge by unencapsulated and encapsulated MDR isolates. (A to D) Survival curves of CD-1 mice immunized 3 times with long-chain O25b-CRM_197_ conjugates: RAC/DMSO (RAC), eTEC single-end (SE), and unconjugated O25b polysaccharide or PBS controls. Mice were challenged i.p. with E. coli O25b strain PFEEC0102 (∼2 × 10^8^ CFU), PFEEC0068 (∼1.55 × 10^8^ CFU), PFEEC0066 (∼5 × 10^6^ CFU), or PFEEC0065 (∼5 × 10^6^ CFU). (E) Survival of CD-1 mice similarly immunized but challenged i.v. with E. coli O25b strain PFEEC0066 (1 × 10^8^ CFU/animal). Ratios indicate the numbers of mice surviving after 72 h over the total number of challenged animals. Asterisks indicate *P* values between control groups (polysaccharide and PBS) and glycoconjugate-vaccinated groups (****, *P* < 0.0001; ***, *P* < 0.001; *, *P* < 0.05; ns, not significant). Ratios indicate the numbers of mice surviving after 72 h over the total number of challenged animals.

Subsequently, vaccine protection experiments were run with all four challenge strains in which the efficacies of monovalent long-chain RAC/DMSO and eTEC lattice conjugates after three 2-μg doses were compared with that of the long-chain single-end conjugate. Negative-control groups were vaccinated with free unconjugated long-chain O25b O antigen or phosphate-buffered saline (PBS) buffer. Results demonstrate that the RAC/DMSO and eTEC lattice conjugates provided >85% protection against i.p. challenge with all four clinical isolates. In contrast, the survival rate of animals in both control groups never exceeded 32% (Fig. 5A to D). The single-end long O-antigen conjugate was less protective than the lattice conjugates when mice were challenged with the hypervirulent O25b:K5 strain, but provided similar levels of protection against challenge with the other three strains. Significantly, three 2-μg doses of the long-chain RAC/DMSO conjugate also protected 75% of mice from lethal i.v. challenge with this hypervirulent strain. In comparison, >65% of animals in the PBS- or unconjugated long-chain O25b O-antigen-treated groups succumbed to the infection in the i.v. challenge model (Fig. 5E). As two vaccine doses generated lower levels of O-antigen-specific IgG and functional antibodies than three doses (Fig. 4), we assessed whether the immune response after two doses would confer protection against this strain. As shown in Fig. S8 in the supplemental material, after only two 2-μg doses of O25b long-chain lattice conjugates, >75% of mice were protected from challenge with the hypervirulent O25b:K5 strain, regardless of conjugation chemistry (RAC/DMSO or eTEC) or presence of AlPO_4_ in the formulation. In these settings, ≤40% of placebo or unconjugated long-chain O25b O-antigen-vaccinated animals survived the lethal challenge. These results indicate that long-chain O25b vaccine lattice conjugates confer protection in mice even with a dosing regimen that elicits intermediate levels of functional antibody.

### Detection of anti-O25b O-antigen antibodies in sera from cynomolgus macaques.

**FIG 6:**
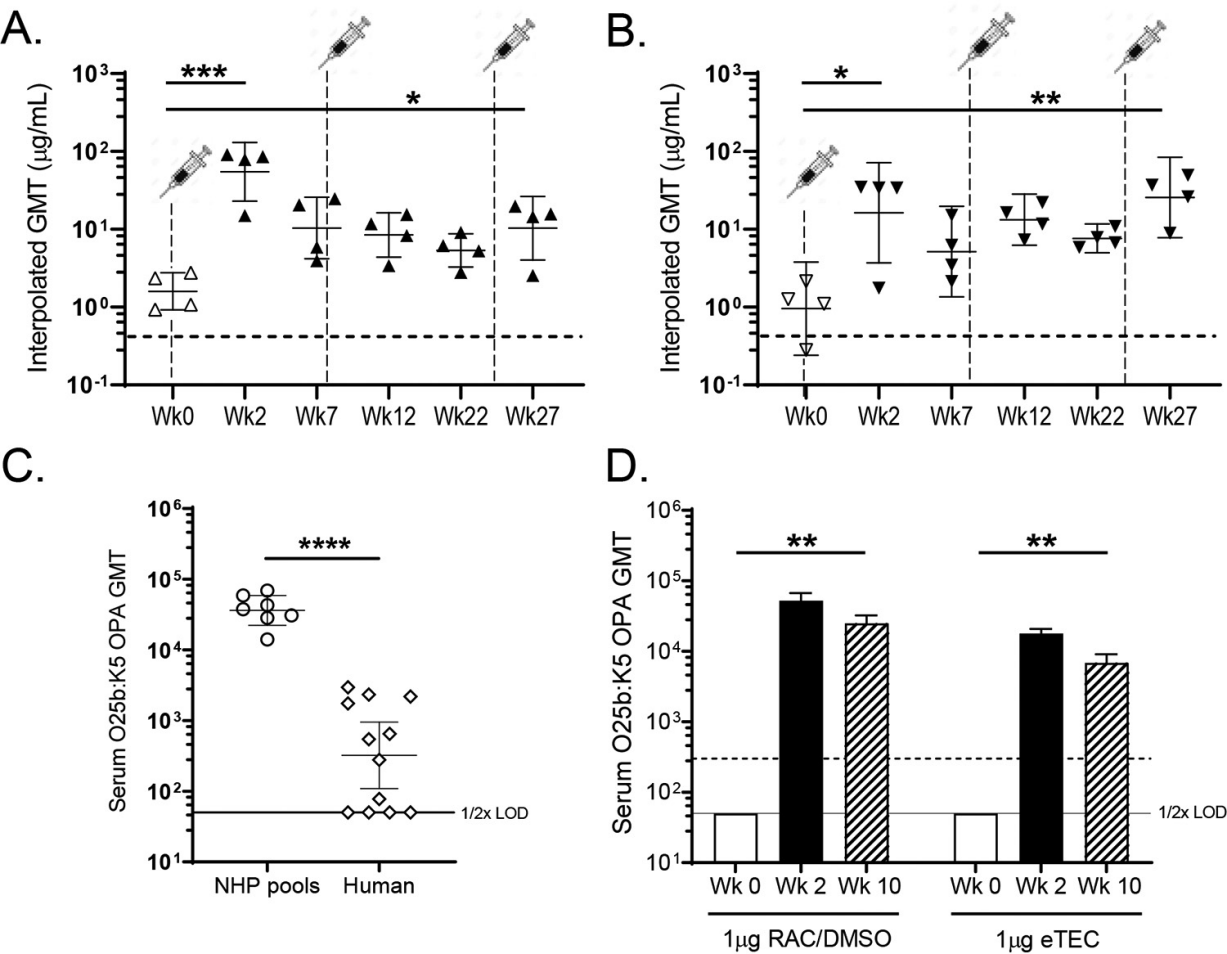
A single dose of O25b RAC or eTEC conjugate is sufficient to elicit robust IgG and OPA responses in cynomolgus macaques. (A and B) Prevaccination (open symbols) and postvaccination (closed symbol) IgG GMT values. (C) O25b OPA activity with strain PFEEC0066(K5) comparing prevaccination NHP serum pools with normal human sera. (D) Analogous postvaccination OPA GMTs of pooled NHP sera after depletion of nonspecific E. coli antibodies with an O25b Δ*waaL* strain derived from isolate PFEEC0102. Adsorption reduces NHP prevaccination OPA titers to below the assay LOD (50 or 1/2× LOD). Dotted lines indicate baseline IgG or OPA titers of sera pooled from 46 unvaccinated human volunteers. ****, *P* < 0.0001; ***, *P* < 0.001; **, *P* < 0.005; *, *P* < 0.05.

To assess the immunogenicity of long-chain RAC/DMSO and eTEC O25b glycoconjugates in NHPs, groups of four cynomolgus macaques were vaccinated intramuscularly (i.m.) with 1 μg or 10 μg of antigen (0.5 mL) at week 0, 8, and 24 time points. Additional groups of three macaques were vaccinated with 1 μg of the RAC/DMSO or eTEC conjugates formulated with 250 μg AlPO_4_. Levels of O25b-specific IgG elicited by these conjugates in the unadjuvanted 1-μg dose groups are shown in Fig. 6A and B). After a single 1-μg dose of either RAC/DMSO or eTEC conjugate, IgG levels rose approximately 10-fold relative to prevaccination levels. Additional vaccinations did not further enhance antigen-specific IgG levels. Groups vaccinated with 10 μg of antigen without adjuvant or 1 μg of antigen with AlPO_4_ showed equivalent responses to groups vaccinated with 1 μg of unadjuvanted glycoconjugate (data not shown). Next, functional responses to 1 μg of unadjuvanted antigen were assessed in OPAs with the O25b:K5 strain. Unexpectedly, 100-fold-higher baseline OPA titers were observed in sera pooled from groups of individual unvaccinated animals than in normal human sera (Fig. 6C). As high levels of O25b-specific IgG were absent from these unvaccinated monkeys, we surmised that non-O-antigen anti-E. coli antibodies resulting from natural exposure might be responsible. To address this possibility, we adsorbed sera pooled from each group with an O25b Δ*waaL* mutant strain lacking detectable surface O antigen. Following this depletion step, the OPA activity of the unvaccinated serum pool was reduced to the assay limit of detection (LOD), while revealing robust O25b-specific functional activity at the week 2 (PD1) and week 10 (PD2) time points (Fig. 6D). Consistent with the corresponding O25b-specific IgG titers, functional OPA titers of vaccinated group serum pools did not increase after the second booster dose.

## DISCUSSION

The rapid emergence of the pandemic invasive O25b MDR STS131 E. coli lineage was described in a systematic longitudinal bioinformatic surveillance study of invasive U.K. E. coli bloodstream infections by the Sanger Institute (27). Their analysis of 1,509 BSI isolates collected from 2001 to 2012 revealed that after appearing in 2003, the MDR O25b ST131 lineage became dominant, while the overall population remained relatively stable. We extend these observations in that MDR O25b ST131 became the most prevalent serotype among both U.S. BSI and global UTI strains collected between 2013 and 2016. These strains are predominantly resistant to fluoroquinolone antibiotics and harbor β-lactamase resistance determinants for cephalosporin β-lactams. A significant subset (33% of BSI strains and 36% of UTI strains) are resistant to front-line oral third-generation cephalosporins (ceftriaxone, ceftazidime, or cefotaxime), which is attributed to the presence of CTX-M-15 and/or OXA1 β-lactamase genes carried by incFIA and or incFIB plasmids (27). This is noteworthy as extended-spectrum cephalosporin resistance increased in U.S. hospitals from 5.46% to 12.97% between 2009 and 2016 (28). In addition, a recent Dutch study found that resistance to third-generation cephalosporins in *Enterobacteriaceae* is a significant risk factor for recurrence in bloodstream infections (29). The shared relative prevalence of the 12 most common O-antigen serotypes between BSI and UTI strains is consistent with the association between strains responsible for sepsis and strains sourced from urinary tract infections (30–33). The distribution of serotypes in our BSI and UTI collections is an important factor in considering the composition of a potential multivalent E. coli O-antigen vaccine to prevent both bloodstream and urinary tract infections. Accordingly, a 12-valent vaccine would provide theoretical coverage of ≥70% of BSI and UTI strains.

The use of vaccines to combat antimicrobial resistance is well described (34, 35). For prevention of invasive MDR E. coli ST131 infections, a highly immunogenic and protective O25b O-antigen glycoconjugate is required. Observations that the serotype O25a or O25b O antigen may be a relatively weak immunogen in both preclinical and clinical studies compared with other O-antigen serotypes (15, 16, 19–21) prompted us to explore conjugate quality attributes such as chain length, degree of cross-linking, and conjugation chemistry to identify the most immunogenic configuration. Using CRM_197_ as carrier protein, we demonstrated in mice that serotype O25b glycoconjugates constructed by intermolecular cross-linking of long-chain O antigens in a high-molecular-weight lattice configuration elicit stronger functional OPA responses than lower-molecular-weight short- or long-chain “sun” conjugates created by single-end directional coupling. As the corresponding antigen-specific IgG responses to these constructs were not significantly different, the results indicated that the potency of the antibody functional response can be improved by intermolecular cross-linking of the O25b O antigen to the CRM_197_ carrier protein. Such higher-molecular-mass CRM_197_ carrier protein lattice conjugates may present a higher density of O25b-specific functional antibody epitopes per carrier protein complex compared with single-end conjugates. In support of this idea is the observation that for the eTEC glycoconjugates, a higher degree of O-antigen activation and cross-linking was associated with improved functional immunogenicity in the OPA (Table S1, Fig. S2). Although IgG or OPA antibody responses to the two distinct lattice conjugates were not significantly different, the greater simplicity and scalability of glycoconjugates made with the RAC/DMSO process compared with the eTEC approach offers a practical advantage for manufacturing. The RAC/DMSO platform chemistry has been successfully used for the production of multivalent CRM_197_ conjugates for the licensed Prevnar13 vaccine and exploratory hexavalent group B streptocococcal capsular polysaccharide vaccine (36).

With regard to protective efficacy in mice, the long-chain RAC/DMSO lattice conjugate was significantly more potent than the long-chain single-end conjugate in protecting against lethal i.p. challenge with a hypervirulent MDR ST131 O25b:K5 isolate. The long-chain lattice conjugate also protected against lethal challenge via the i.v. route of infection. We find these results encouraging, as mechanisms of immune clearance of bacteria following i.p. versus i.v. administration are likely to differ; lethal i.v. administration requires an approximately 50-fold-higher inoculum of bacteria, and mitigation likely involves neutralizing associated LPS endotoxin together with clearance by liver macrophages (37). An additional practical advantage of engineered long-chain O antigens compared with native short-chain O-antigen polysaccharides is compatibility with established bioprocess purification and chemical conjugation methods used for licensed capsular polysaccharide antigen-based pneumococcal vaccines (36). In addition, recombinant long-chain O antigens are easier to separate from low-molecular-weight contaminants by diafiltration, and the efficiency of chemical cross-linking to generate lattice conjugates is greater than for native short-chain O-antigen polysaccharides.

In these preclinical studies, we observed differences in the immunogenicity of O25b glycoconjugates between species. Mice and rabbits required multiple booster doses to achieve peak antibody titers in the absence of adjuvant. In contrast, a second booster dose of glycoconjugate in NHPs did not increase antigen-specific IgG or bactericidal antibody responses (Fig. 6A and B). We speculate that high levels of preexisting E. coli antibodies in normal sera of NHPs may prime animals for subsequent exposure to pathogenic E. coli. Indeed, we observed high levels of non-O-antigen-specific functional antibodies in prevaccination sera, which required removal (by adsorption with an O-antigen null mutant) to detect underlying O25b-specific functional responses (Fig. 6D). In contrast baseline OPA titers of normal human sera were substantially lower (Fig. 6C). Another difference we observed between mouse and NHP antibody responses to the long-chain lattice glycoconjugates is that while the AlPO_4_ formulation improved OPA responses in mice, it had no impact on immunogenicity in NHPs. Clearly, clinical studies will be required to assess the boostability and formulation of lattice glycoconjugate antigens in human subjects.

In summary, using the serotype O25b O antigen as a test case, we developed a conjugation strategy that combines increased polysaccharide chain length and intermolecular CRM_197_ carrier protein cross-linking to augment the functional activity of elicited antibodies in *in vitro* OPAs and in mouse lethal challenge models with hypervirulent MDR ST131 E. coli. As chain length enhancement can, in theory, be applied to the majority of E. coli O antigens synthesized via the Wzx/Wzy pathway (reviewed in reference 38), we expect this will spur the development of a broadly protective multivalent conjugate vaccine to prevent invasive E. coli infections. For serotypes O8 and O9, where O antigens are synthesized via the ABC transporter pathway, directional single-end conjugation of short-chain O antigens to CRM_197_ will be required.

## MATERIALS AND METHODS

### Bacterial strains.

E. coli clinical BSI and UTI isolates were selected in an unbiased manner from the Pfizer-sponsored Antimicrobial Testing Leadership and Surveillance (ATLAS) collection, maintained by the International Health Management Associates (IHMA) clinical lab. Three additional serotype O25b isolates (PFEEC0065, PFEEC0066, and PFEEC0068) were obtained separately from IHMA based on their multidrug resistance properties. Strains were genotypically characterized by whole-genome sequencing (WGS) using the Miseq platform (Illumina). DNA fragment libraries were prepared using a Nextera XT DNA library preparation kit (Illumina). WGS data were used to generate multilocus sequence type (MLST) information using the seven-locus E. coli scheme integrated into the BIGSdb platform (39, 40). Embedded *in silico* serotyping algorithms were used to predict O-antigen serotype, LPS oligosaccharide core type, and FimH type (41, 42). Two additional BSI strains from the Wyeth Tygacil collection isolated in 2003 were modified for O-antigen production. E. coli *wzzB* or *waaL* genes were removed using the λ-Red-mediated homologous recombination system as previously described (43). A *wzzB* deletion was first introduced into strain PFEEC0100, an ST45 isolate expressing LPS with O25b O antigen and the R1 LPS core oligosaccharide. To generate *wzzB* and *waaL* knockouts in ESBL strain PFEEC0102, which expresses LPS with O antigen attached to the K12 LPS core oligosaccharide, it was necessary to replace the *amp* selectable marker in recombineering plasmids pKD43 (λ-Red) and pCP20 (FLP recombinase) with *tet*. A PCR fragment containing the Salmonella *fepE* gene and promoter was amplified from genomic DNA prepared from S. enterica serovar Typhimurium strain LT2 (ATCC 700720), using primers described by Murray et al. (24). Analogous *wzzB* or *fepE* gene fragments were similarly amplified from E. coli strains, cloned into the PCR Blunt II TOPO vector (Invitrogen), and introduced into *wzzB* host strains. LPS was extracted from bacteria using a phenol-based kit (BulldogBio).

### Animal immunogenicity and challenge models.

To assess the virulence of O25b E. coli clinical isolates *in vivo*, naive CD-1 mice (12 to 14 weeks of age; Charles River Laboratories) were challenged with various doses and strains diluted in PBS and injected i.p. (0.25 mL) or i.v. (0.2 mL). To evaluate the efficacy of O25b candidate vaccines, immunized CD-1 mice were challenged i.p. (0.25 mL) with a lethal dose of each O25b strain, which was calibrated in separate dose ranging studies. Animals were monitored every 2 to 3 h during the first 8 h and again during the 20- to 32-h window period postchallenge. Observations continued as needed up to 72 h postchallenge. New Zealand White female rabbits were vaccinated s.c. with 10 μg/dose (0.5 mL) at weeks 0, 6, and 10 with 10 μg of glycoconjugate and 20 μg of QS21 adjuvant Female CD-1 mice (6 to 8 weeks of age; Charles River Laboratories) were immunized with 0.2-μg or 2-μg doses by s.c. injection (0.1 mL). Animals were bled (submandibular) 1 to 2 weeks after each immunization and exsanguinated at the end of the study. Endotoxin levels for all antigen formulations were below 0.1 EU/dose/animal (below 0.05 EU/μg). All female cynomolgus macaques (Macaca fasicularis; age range, 7 to 15 years; weight range, 3.6 to 7.2 kg) were housed in standard quad caging at Pfizer (Pearl River, NY). Animals were provided with unrestricted access to water and fed a standard diet. NHPs were vaccinated i.m. (0.5 mL) with either 1 μg or 10 μg of long-chain RAC/DMSO or eTEC O25b glycoconjugate at weeks 0, 8, and 24. Following the same immunization schedule, additional animals received 1 μg of the RAC/DMSO or eTEC O25b conjugates adjuvanted with 250 μg of AlPO_4_. Blood samples were collected weekly or biweekly over a 27-week period and then monthly until week 57. Blood was withdrawn in 3.5-mL serum tubes at each time point and spun in a centrifuge at 3,000 rpm for 10 min. The serum fractions were collected and stored in cryovials. Animal studies were conducted according to Pfizer local and global Institutional Animal Care and Use Committee (IACUC) guidelines at an Association for Assessment and Accreditation of Laboratory Animal Care (AAALAC) International-accredited facility.

### O-antigen purification.

The fermentation broth was treated with acetic acid to a final concentration of 1 to 2% (final pH of 4.1). The extraction of O antigen and delipidation were achieved by heating the acid-treated broth to 100°C for 2 h. After acid hydrolysis, the batch was cooled to ambient temperature, and 14% NH_4_OH was added to a final pH of 6.1. The neutralized broth was centrifuged, and the centrate was collected. To the centrate, 5 M CaCl_2_ stock solution in sodium phosphate was added to a final CaCl_2_ concentration of 0.1 to 0.2 M, and the resulting slurry was incubated for 30 min at room temperature (RT). The solids were removed by centrifugation, and the centrate was concentrated 10- to 12-fold using a 10-kDa molecular-weight-cutoff (MWCO) Sartocon Hydrosart membrane (Sartorius), followed by two diafiltrations against 20 mM citrate (pH 6.0) and water, respectively, with 10 diavolumes for each diafiltration. The retentate that contained the O antigen was then purified using a carbon filter (3M CUNO R32SP). The carbon filtrate was diluted 1:1 (vol/vol) with 4.0 M ammonium sulfate [(NH_4_)_2_SO_4_]. The final (NH_4_)_2_SO_4_ concentration was 2 M. The (NH_4_)_2_SO_4_-treated carbon filtrate was further purified using a Sartobind phenyl membrane (Sartorius) at the loading capacity of 55 mg of O antigen per mL of membrane volume with 2 M (NH_4_)_2_SO_4_ as the running buffer. The O antigen was collected in the flowthrough. For the long O antigen, the HIC filtrate was concentrated to the desired concentration level and then buffer exchanged against water (20 diavolumes) using a 5-kDa MWCO Sartocon Hydrosart membrane from Sartorius. For the short (native) O-antigen polysaccharide, the MWCO was further reduced to enhance yield.

### Structural analysis via NMR spectroscopy.

Lyophilized O25b O-antigen polysaccharide was weighed and dissolved in D_2_O. The sample was incubated for 20 min in a sonicated water bath at 50°C. The resuspended polysaccharide was spun down to remove any insoluble aggregates. The sample was transferred into the 5-mm nuclear magnetic resonance (NMR) tube for data collection. All data were collected at 70°C using Bruker 600-MHz spectrometer equipped with a TCI cryoprobe. The following one-dimensional (1D) and 2D NMR data were collected for complete structural analysis: 1D ^1^H (d1 5s; 32k point), 2D ^1^H-^13^C heteronuclear single quantum coherence (HSQC), ^1^H-^13^C HMBC, ^1^H-^13^C HSQC correlation spectroscopy (COSY), ^1^H-^13^C HSQC total correlation spectroscopy (TOCSY) with a mixing time of 120 ms, and multiplicity-edited ^1^H-^13^C HSQC. The ^1^J_CH_ correlations were determined using the ^1^H-^13^C CLIP-HSQC experiments. All 2D experiments were carried out using 2,024 and 256 data points in the ^1^H and ^13^C dimensions, respectively. The NMR data were processed using NvX and analyzed using NMRViewJ (44) software. The chemical shift difference was plotted using the equation

$$\text{CSD = }\sqrt{{\text{δH}}^{2}+({0.3\times \delta \text{C}}^{2})}$$

where, δH and δC are the proton and carbon chemical shifts, respectively.

### O-antigen conjugation to CRM_197_.

Long-chain O25b polysaccharide-CRM_197_ conjugates were initially produced using periodate oxidation followed by conjugation using reductive amination chemistry (RAC). Conjugate variants with three activation levels (low, medium, and high) were produced by varying the oxidation levels. Conjugates were produced by reacting the lyophilized activated polysaccharides with lyophilized CRM_197_, reconstituted in DMSO medium, using sodium cyanoborohydride as the reducing agent. Conjugation reactions were carried out at 23°C for 24 h, followed by capping using sodium borohydride for 3 h. Following the conjugation quenching step, conjugates were purified by ultrafiltration/diafiltration with 100-kDa MWCO regenerated cellulose membrane, using 5 mM succinate–0.9% NaCl (pH 6.0). Final filtration of the conjugates was performed using a 0.22-μm-pore membrane. A second type of conjugate was generated with the use of a bivalent, heterobifunctional linker referred to as a (2-((2-oxoethyl)thio)ethyl)carbamate (eTEC) spacer. In this case, the O-antigen polysaccharide was covalently linked to the eTEC spacer through a carbamate linkage, while the carrier protein was covalently linked to the eTEC spacer through an amide linkage. For single-end directional coupling, the O antigen went through activation/reduction via a linker, dithio-propionic dihydrazide, to insert an amine thiol group at the KDO carbonyl present at the polysaccharide reducing end. The activated O-antigen polysaccharide was then conjugated to bromo-CRM_197_.

### Detection of antigen-specific IgG in sera.

For detection of O25b-specific IgG, long O-antigen polysaccharide was covalently conjugated to poly-l-lysine with CDAP (1-cyano-4-dimethylaminopyridinium), and the derived poly-l-lysine conjugate was covalently coupled to magnetic carboxy bead microspheres (Magplex; Luminex) with EDC/NHS [1-ethyl-3-(3-dimethylamino) propyl carbodiimide/*N*-hydroxysuccinimide] (Thermo Fisher). Beads were incubated with serially diluted individual mouse sera or control MAb with shaking at 4°C for 18 h. After washing, bound serotype-specific IgG was detected with a phycoerythrin (PE)-conjugated goat anti-mouse total IgG secondary antibody (Jackson ImmunoResearch) after 60 min of RT incubation. Microplates were read on a FlexMap 3D instrument (Bio-Rad). A serotype-specific IgG MAb was used as an internal standard to quantify IgG levels. A standard curve plot for the MAb titration yielded a linear slope profile across a 10^3^ range of serum dilutions (log luminescence versus log serum dilution).

### Flow cytometry strain characterization.

O-antigen expression was measured by flow cytometry (Satorius, iQue). Bacteria were fixed in 4% paraformaldehyde and stained with primary antibodies (O25b-specific MAb, preimmunized sera, and/or postvaccinated rabbit sera), and O antigens were detected with PE-conjugated anti-rabbit polyclonal or anti-human secondary MAbs.

### Serum opsonophagocytic assays.

Bacterial stocks were prepared by growing bacteria in Dulbecco’s modified Eagle’s medium (DMEM) to an optical density at 600 nm (OD_600_) of between 0.5 and 1.0, and glycerol was added to a final concentration of 20% prior to freezing. For unencapsulated strains, pretitered thawed bacteria were diluted to 1 × 10^5^ CFU/mL in OPA buffer (Hanks balanced salt solution [Life Technologies], 0.1% gelatin), and 20 μL (10^3^ CFU) of the bacterial suspension was opsonized with 10 μL of serially diluted serum for 30 min at RT in a 384-well tissue culture microplate. Subsequently, 10 μL of 2.5% complement (baby rabbit serum; Pel-Freez) and 10 μL of HL60 cells (at 100:1 ratio) were added to each well, and the mixture was shaken at 2,000 rpm for 45 to 60 min at 37°C in a 5% CO_2_ incubator. For encapsulated strains, bacteria were directly combined with complement and HL60s without the preopsonization step and shaken at 2,000 rpm for 60 min at 37°C under 5% CO_2_. In this case, 4% baby rabbit complement and HL60 cells at a 100:1 bacterial ratio were used. After the incubation, 10 μL of each 50-μL reaction mixture was transferred into the corresponding wells of a prewetted 384-well Millipore MultiScreen HTS HV filter plate containing 50 μL water/well. After vacuum filtering the liquid, 50 μL of 50% DMEM was applied and filtered, and the plate was incubated overnight at 37°C in a sealed zip-lock bag. The next day, the colonies were enumerated after staining with Coomassie dye using an ImmunoSpot analyzer and Immunocapture software (Cellular Technology, Ltd.). To establish the specificity of OPA activity, immune sera were preincubated with 20 μg/mL of the homologous serotype purified O antigen prior to the opsonization step. The OPA included control reactions without HL60 cells or complement, to demonstrate dependence of any observed killing on these components. Individual serum OPA titers were calculated using variable-slope curve fitting (Excel). Combined data were plotted using GraphPad Prism to generate GMTs and associated *P* values for significance (one-way analysis of variance [ANOVA] with log-transformed data).

### Statistical analyses.

Statistical differences in animal survival plots were determined using a log rank test (the Mantel-Cox test), and statistical differences of immunogenicity data were determined using log-transformed data followed by analysis by unpaired Student *t* tests with Welch correction of data (both are included in GraphPad Prism 7.02).

### Data availability.

Primary nucleotide sequence data for the 551 E. coli isolates from this study will be deposited in the NCBI Sequence Read Archive (BioProject no. PRJNA804716).
