# Supplementary material for: Preclinical Immunogenicity and Efficacy of Optimized O25b O-Antigen Glycoconjugates To Prevent MDR ST131 E. coli Infections
Source: Infect Immun. 2022 Mar 21;90(4):e00022-22. doi: 10.1128/iai.00022-22 (PMC9022517; doi:10.1128/iai.00022-22)
Supplement: Supplemental file 1 — supplemental material. Download iai.00022-22-s0001.pdf, PDF file, 0.7 MB [file iai.00022-22-s0001.pdf]

Fig. S1

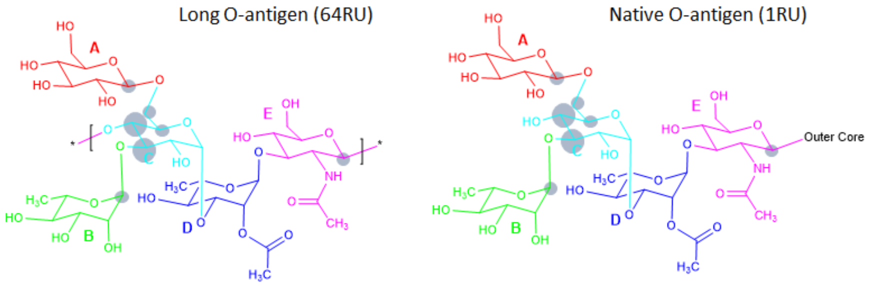

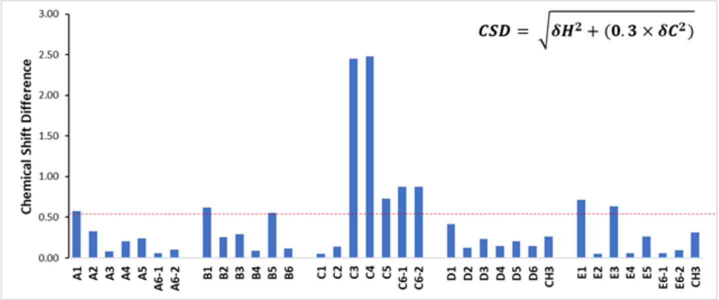

		Present Assignment		Literature Assignment	
		1H (ppm)	13C (ppm)	1H (ppm)	13C (ppm)
β-D-Glc*p* (A)	A1	4.53	102.4	4.5	103.5
	A2	3.34	73.4	3.31	74
	A3	3.52	76.4	3.5	76.5
	A4	3.42	70.1	3.39	70.5
	A5	3.46	76.4	3.45	76.8
	A6-1	3.92	61.4	3.92	61.5
	A6-2	3.75	61.3	3.72	61.5
α-L-Rha*p* (B)	B1	5.22	100.8	5.09	101.9
	B2	4.02	70.6	4.04	71.1
	B3	3.88	70.5	3.78	71
	B4	3.46	72.6	3.44	72.8
	B5	4.39	68.8	4.02	69.6
	B6	1.33	17.1	1.25	17.3
α-D-Glc*p* (C)	C1	4.93	95.4	4.95	95.5
	C2	3.68	72.3	3.66	72.1
	C3	3.90	76.2	3.79	80.7
	C4	3.89	72.9	3.61	68.4
	C5	4.04	70.4	4.05	71.7
	C6-1	4.14	67.3	4.14	68.9
	C6-2	3.78	67.3	3.89	68.9
α-L-Rha*p* 2OAc (D)	D1	4.93	98.8	4.92	99.6
	D2	5.18	69.1	5.2	68.9
	D3	3.94	73.7	3.99	73.3
	D4	3.60	70.9	3.62	71.2
	D5	4.05	69.1	4.1	69.5
	D6	1.28	16.9	1.28	17.2
	CH_3	2.14	20.6	2.13	21.1
	CO		173.5		173.8
β-D-Glc*p* NAc (E)	E1	4.62	100.0	4.59	101.3
	E2	3.81	56.1	3.84	56.2
	E3	3.66	81.9	3.59	83.1
	E4	3.50	69.2	3.55	69.2
	E5	3.43	76.3	3.45	76.8
	E6-1	3.94	61.6	3.93	61.5
	E6-2	3.82	61.6	3.76	61.5
	CH_3	2.01	22.6	2.05	23.2
	CO		174.6		175.5

Table S1.

**Conjugate:**	Short-chain single-end	Long-chain single-end	Long-chain RAC/DMSO	Long-chain eTEC	Long-chain eTEC	Long-chain eTEC
Saccharide MW (kDa)	14	48	51	48	48	48
Activation	6.6% SH†	2.4% SH†	DO:18*	4% SH†	10% SH†	17% SH†
Yield (%)	28	26	82	32	56	92
SPR ratio‡	0.7	0.9	1	0.7	0.8	1.2
Free Saccharide (%)	< 5	<10	12.1	<15	<10	<13
Conjugate MW (kDa)	380	1423	5365	1220	1258	2736
Saccharide conc. (mg/mL)	0.45	0.44	0.75	0.37	0.38	0.87
Endotoxin (EU/mg)	0.01	0.02	0.01	0.08	0.08	0.01

†Degree of sulfide activation; *Degree of periodate oxidation; ‡ Saccharide to protein ratio (by mass); Buffer matrix is 5 mM Succ/Saline, pH 6.0.

Fig. S2

A.

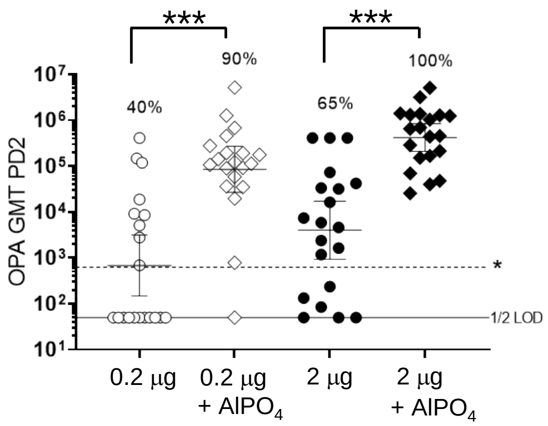

RAC/DMSO Conjugate	Dose (µg)	OPA GMT (95% CI)	% Responder Rate
Wk0	none	629	NA
RAC	0.2	683 (155, 3019)	40
RAC+$AlPO_4$	0.2	84,706 (27410, 261776)	90
RAC	2	8,296 (967, 16556)	65
RAC+$AlPO_4$	2	421,392 (213868, 830286)	100

B.

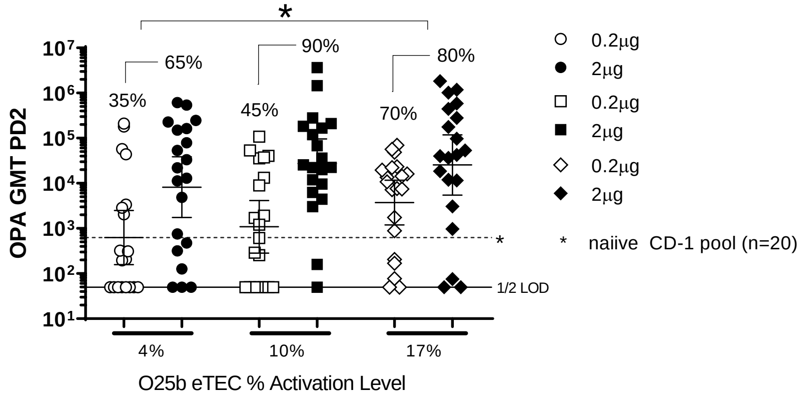

eTEC Activation Level	Dose (µg)	OPA GMT (95% CI)	% Responder Rate
0 (wk0)	none	629	NA
4%	0.2	628 (163, 2419)	35
4%	2	8,185 (1809, 37036)	65
10%	0.2	1085 (294, 4008)	45
10%	2	27,368 (8017, 93431)	90
17%	0.2	3,734 (1228, 11352)	70
17%	2	25,462 (5689,113955)	80

FIG S2 OPA titers and responder rates generated with MDR strain MDR PFEEC0068 at the sub-peak PD2 timepoint reveal benefits of $AlPO_4$ formulation with the RAC/DMSO glycoconjugate and allows optimization of the eTEC conjugation chemistry. A) Impact of $AlPO_4$ on RAC/DMSO conjugate immunogenicity. B) Impact of degree of linker sulfhydryl activation on eTEC conjugate immunogenicity. Shown are OPA responses at low dose (open symbols) and high dose (closed symbols). *** $p < 0.001$; * $p < 0.05$.

Fig. S3

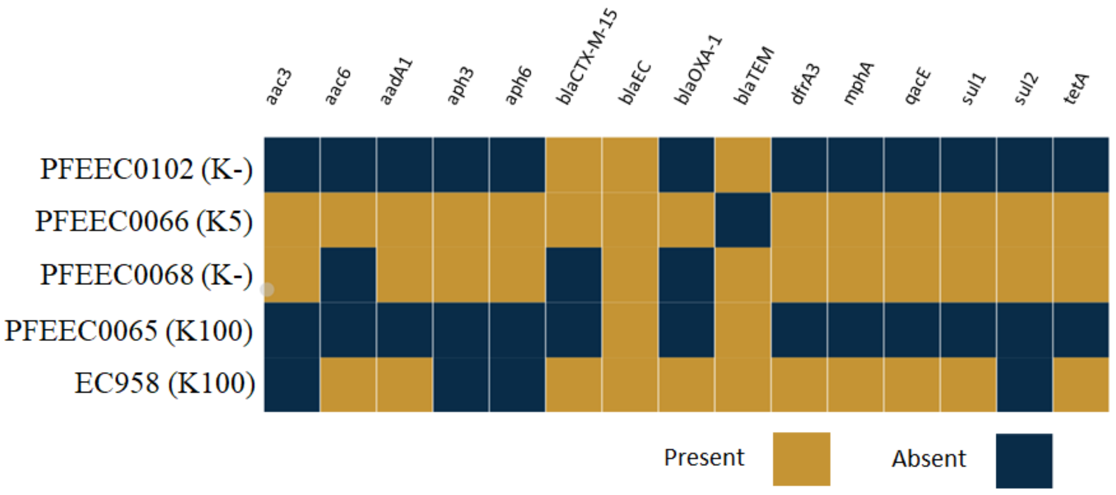

FIG S3. Summary of the absence and presence of the MDR genes in *E. coli* ST131 blood isolates. Genes are associated with resistance to aminoglycosides (*aac*, *aad*, *aph*), beta-lactamases (*bla*), antifolate-sulfur combinations (*dfr, sul1, sul2*) macrolide (*mphA*) and tetracycline (*tetA*). In *E.coli* ST131 these resistance determinants are carried on IncF1A, IncF1B plasmids. The efflux pump gene (*qacE*) contributes resistance to disinfectants. Each row is a *E. coli* isolate and each column represent a MDR gene. A blue-color filled cell represents the given MDR gene is absent in the genome of the isolate whereas a yellow-color filled cell represents the given MDR gene is present as determined by Ariba and NCBI Blast using the Bacterial Antimicrobial Resistance Reference Gene Database (PRJNA313047).

Fig. S4

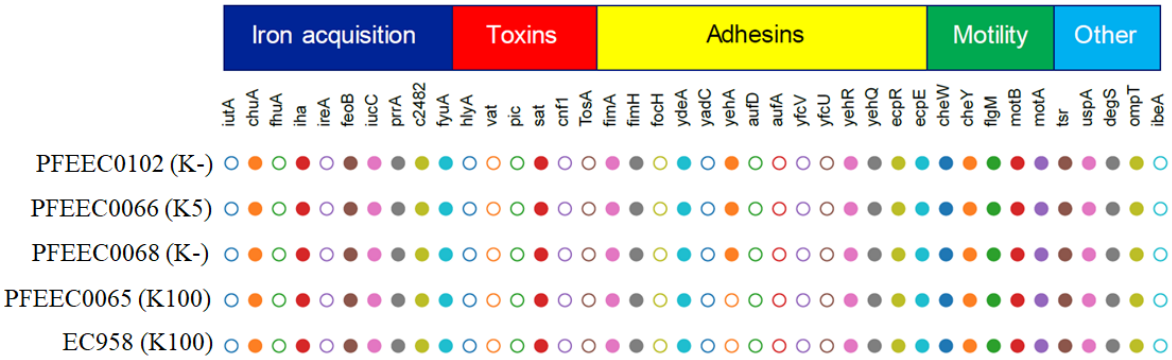

FIG S4. Presence/absence of UPEC virulence genes in *E. coli* blood isolates. MDR BSI isolates show similar profile of UPEC virulence factor genes. BLAST analysis was performed with a list of proteins previously implicated in UTIs from in vitro or in vivo studies (Schreiber 2017). To define presence (filled circle) or absence (open circle), a threshold of 90% similarity was applied and E-value <0.05.

Fig. S5

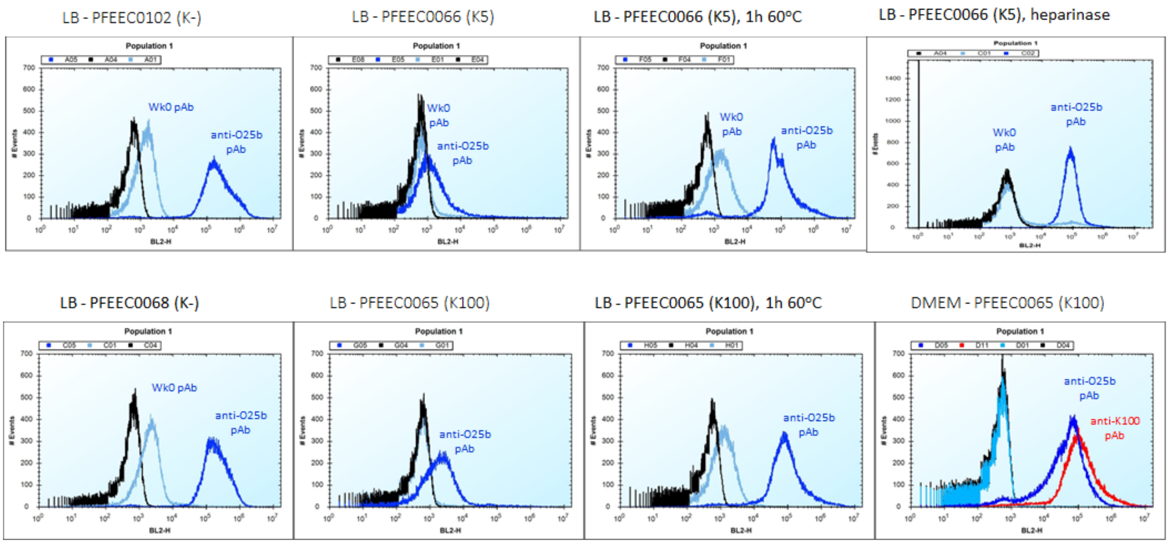

FIG S5. K-capsule masks O-antigen on bacteria grown in LB media. Bacteria were stained with rabbit anti-O25b polyclonal antibodies (pre-immune “wk0”, light blue; post-immune serum, dark blue) or K100-specific capsule typing serum from Statens Institute (red). Strain identities and K-capsule types are indicated. Loss of capsule in LB-grown cells and exposure of O-antigen was demonstrated by heat treatment. Presence of K5 capsule (heparosan) was inferred by the ability to detect O-antigen following heparinase digestion.

Fig. S6

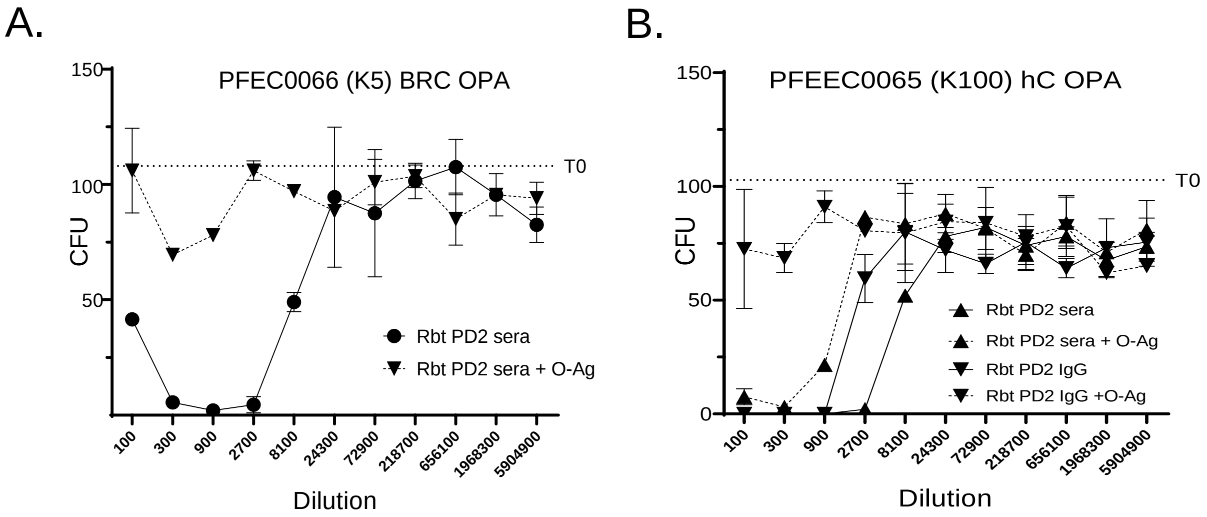

FIG S6. Rabbit antibodies elicited by the O25b RAC/DMSO conjugate kill encapsulated strains in OPAs. A) hypervirulent K5 strain, B) K100 strain. BRC, baby rabbit complement. hC, antibody-depleted human complement. O-Ag, unconjugated O-antigen used to adsorb O-antigen specific antibodies. T0 – time zero cfu level is indicated by the horizontal dotted line. Post-dose two (PD2) rabbit immune sera were evaluated. In the K100 strain assay, OPA activity of the immune serum was partially depleted by antigen, while the activity of affinity purified serum IgG was completely adsorbed by antigen. The ratio of HL60 effector cells to bacteria in both assays was 100:1.

Fig. S7

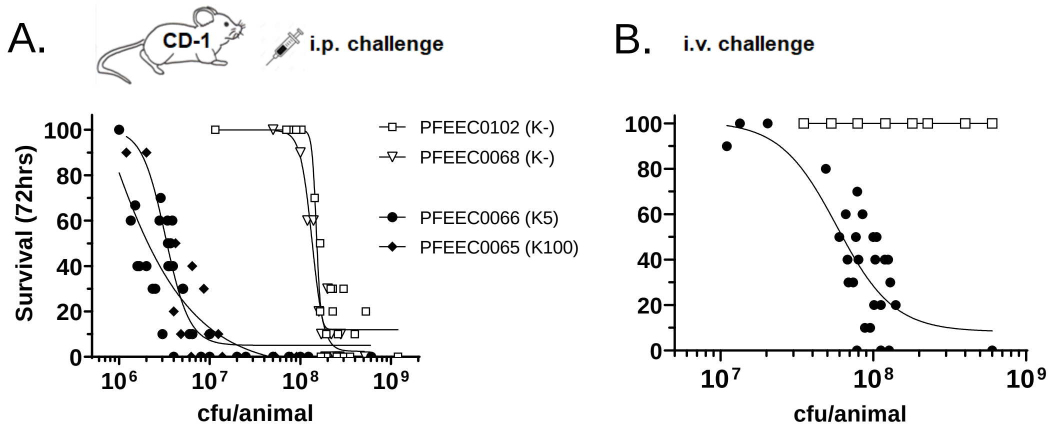

FIG S7. Encapsulated O25b blood isolates are more virulent than unencapsulated strains. A) survival of CD-1 mice following i.p. challenge with bacteria at 72h post-infection. Each symbol represents a group of 10 mice challenged with the indicated inoculum of bacteria (cfu/animal). B) survival after i.v. challenge with the hypervirulent K5 strain PFEEC0066 (closed circles) and unencapsulated strain PFEEC0102 (open squares).

Fig. S8

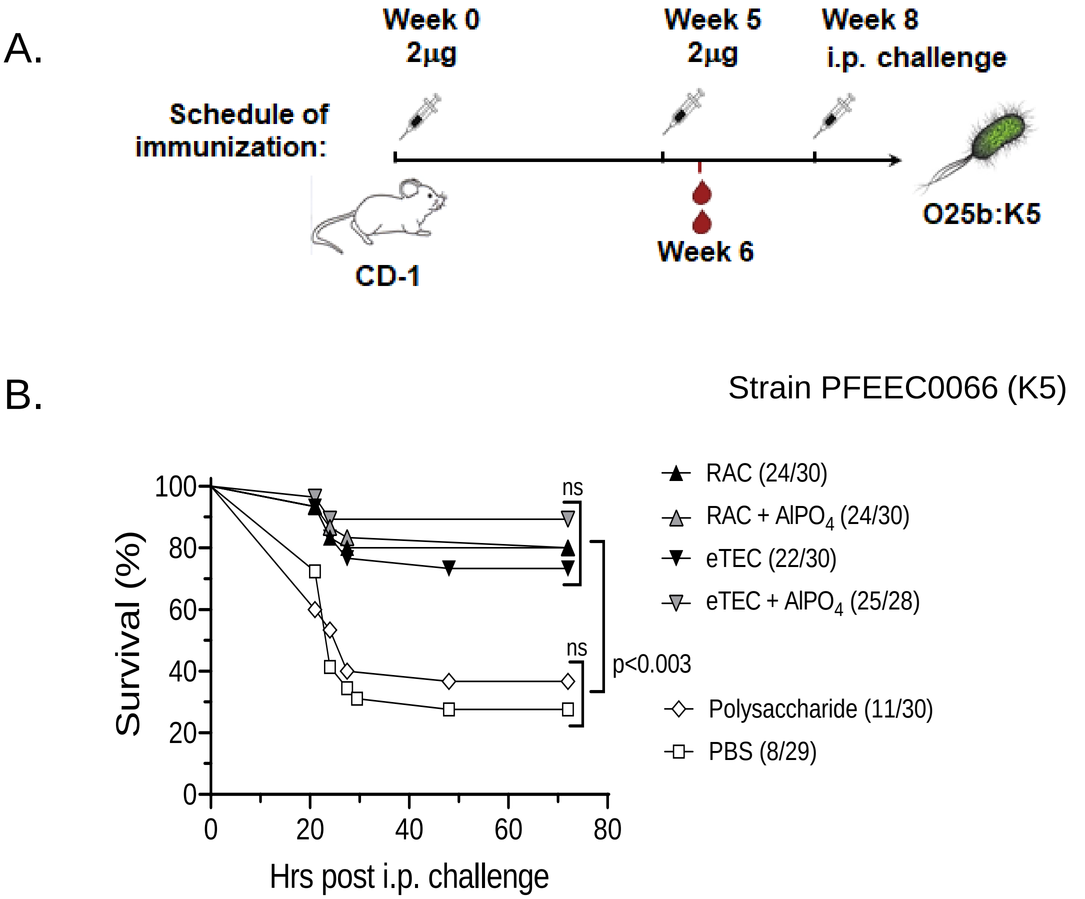

FIG S8. Two vaccine doses are sufficient to protect against lethal challenge with hypervirulent O25b:K5 PFEEC0066. (A) Schematic of the vaccination schedule, dosing and challenge timing. (B) Survival curves of groups of 30 CD-1 mice immunized twice either with long chain O25b-CRM_{197} RAC/DMSO (RAC), eTEC (eTEC), unconjugated O25b polysaccharide or PBS and i.p. challenged with PFEEC0066 (~$5x10^6$ CFU/animal). Ratios indicate the numbers of mice surviving after 72 hrs and the total number of challenged animals.
